# Effect of Combination Use of Aqueous Humor Secretion Inhibitor Eye Drops on Aflibercept Level: A Preliminary Analysis

**DOI:** 10.1167/tvst.14.2.21

**Published:** 2025-02-20

**Authors:** Satoru Inoda, Hidenori Takahashi, Ryota Takahashi, Yuto Hashimoto, Hana Yoshida, Hironori Takahashi, Yujiro Fujino, Kenichi Aizawa, Hidetoshi Kawashima, Yasuo Yanagi

**Affiliations:** 1 Department of Ophthalmology, Jichi Medical University, Shimotsuke-shi, Tochigi, Japan; 2 Department of Ophthalmology, Japan Community Healthcare Organization Tokyo Shinjuku Medical Center, Shinjuku-ku, Tokyo, Japan; 3 Division of Clinical Pharmacology, Department of Pharmacology, Jichi Medical University, Tochigi, Japan; 4 Clinical Pharmacology Center, Jichi Medical University Hospital, Tochigi, Japan; 5 Department of Ophthalmology and Micro-Technology, Yokohama City University, Yokohama, Japan; 6 Retina Research Group, Singapore Eye Research Institute, Singapore Eye-ACP, Duke-NUS Medical School, National University of Singapore, Singapore

**Keywords:** neovascular age-related macular degeneration (nAMD), aqueous humor (AH), eye drops, pharmacokinetics, aflibercept

## Abstract

**Purpose:**

The purpose of this study was to investigate the association between aqueous humor (AH) suppressant eye drops and the concentration of aflibercept at 1 month after intravitreal aflibercept (IVA).

**Methods:**

This retrospective study included 17 eyes of 17 patients with neovascular age-related macular degeneration (nAMD) who used eye drops for their glaucoma and received their first IVA at 2 centers between July 2013 and November 2020. As controls, we enrolled 40 age-, sex-, and axial length-matched eyes of 40 patients with nAMD who were not using any medication that would affect AH circulation. AH was collected 1 month after the first IVA, and aflibercept levels were measured by enzyme-linked immunosorbent assay and were compared between controls and patients using the Kruskal-Wallis test and Dunn's test. The drugs were categorized into two groups based on their mechanism of action on the AH: outflow drugs (e.g. prostaglandin analog) and inflow drugs (e.g. carbonic anhydrase inhibitor, beta-blockers, and alpha-2 agonists).

**Results:**

Mean (interquartile range [IQR]) aflibercept levels in the AH in controls and in patients who used outflow and inflow drugs were 6.83 (IQR = 1.94–10.34), 9.93 (IQR = 2.58–17.44), and 15.95 (IQR = 7.20–22.57) µg/mL, respectively. Aflibercept levels in the AH were significantly higher in inflow drugs users (*P* = 0.0085).

**Conclusions:**

Aflibercept levels in the AH 1 month after the first IVA were higher in patients using eye drops that reduce AH secretion than in controls.

**Translational Relevance:**

Our results, together with previous studies in animals, imply that combination use of these eye drops might extend the half-life of intravitreally injected drugs.

## Introduction

Since their introduction, anti-vascular endothelial growth factor (VEGF) agents have shown proven effectiveness in the treatment of neovascular age-related macular degeneration (nAMD). The standard of care is generally the treat-and-extend regimen, which maintains efficacy while reducing burden. Despite the favorable effects of anti-VEGF agents, anti-VEGF therapy entails considerable patient burden due to the need for regular intravitreal injections. Newly launched anti-VEGF agents have been designed and developed with the aim of extending their half-life and reducing the frequency of injections. A small VEGF-binding molecule such as brolucizumab (26 kilodalton [kDa]) allows for a higher clinical dose in the same volume that can be safely injected intravitreally, potentially prolonging the clinical activity compared with larger molecules such as bevacizumab (149 kDa).^1^ The TENAYA and LUCENE studies showed that the effect of faricimab, which targets not only VEGF but also angiopoietin (Ang)-2, appears to be prolonged compared to aflibercept in terms of maintaining best-corrected visual acuity.^2^

The pharmacokinetics of anti-VEGF agents have been studied using a variety of approaches, including in vivo experiments in rabbits following either intravenous or intravitreal injections. Anti-VEGF agents injected into the eye are not normally metabolized or degraded but are primarily cleared from the vitreous cavity by the anterior pathway.^3^^–^^7^ This means that anti-VEGF drugs passively diffuse into the aqueous humor (AH) and are released into the systemic circulation. The circulation of the AH is controlled by the balance between its secretion and reabsorption, with reabsorption being a passive process. Among eye drops, those for glaucoma lower the intraocular pressure by affecting AH circulation, and some, in particular, inhibit AH secretion and circulation. For example, carbonic anhydrase inhibitors (CAIs), beta-blockers, and alpha-2 agonists are widely used to lower intraocular pressure by reducing AH secretion, whereas prostaglandin analogues (PGAs) and alpha-2 agonists are also used to reduce resistance to AH outflow.^8^^,^^9^

The purpose of this study was to test our hypothesis that, given that anti-VEGF drugs are passively cleared from the eye, slower AH turnover could potentially lead to delayed clearance of anti-VEGF drugs, thereby prolonging their half-life.

## Methods

### Ethical Approval and Consent

This case-control study had a two-center retrospective design. The protocol was approved by the institutional review boards of Jichi Medical University (JICHI20-127) and the Japan Community Healthcare Organization Tokyo Shinjuku Medical Center (CU20-R007, H22-3) and adhered to the tenets of the 1964 Declaration of Helsinki and its later amendments or comparable ethical standards. The study procedures followed our institutional guidelines, and all patients provided oral informed consent before the procedures were performed.

### Procedure

This retrospective study included 17 eyes of 17 patients with nAMD who used eye drops for glaucoma and were treated with the first aflibercept 2 mg injection at 1 of the 2 centers between July 2013 and November 2020. As controls, we used 40 age-matched eyes of 40 patients with nAMD who did not use any eye drops or medication that would affect AH circulation and who were also treated with the first aflibercept 2 mg injection.

We collected approximately 0.2 mL of AH into a disposable syringe just prior to the second aflibercept injection. The liquid was immediately transferred to a sterile tube (ProteoSave; Sumitomo Bakelite Co., Ltd., Tokyo, Japan) and stored at −80°C until measurement.

AH samples used for the measurements were taken 1 month after the first injection. Aflibercept concentrations were measured using the Aflibercept ELISA (enzyme-linked immunosorbent assay) Kit (IG-AA115; AybayTech Biotechnology Ltd., Ankara, Turkey).

### Patients and Controls

Inclusion criteria were as follows: patients with nAMD with glaucoma who received the first aflibercept injection without other ocular diseases and had no medical history of any glaucoma surgery. Controls had nAMD without other ocular diseases. For the control group, we considered the baseline clinical characteristics (age, sex, axial length, time from aflibercept injection to AH sampling, and presence or absence of vitreous) as potential confounders responsible for selection bias. Participants were included if the patient received an aflibercept injection between 28 and 35 days before the AH sampling.

Then, from 316 patients (median age = 75 years; 69% men; median axial length = 23.4 mm), we excluded patients who had undergone vitrectomy. Next, we selected 17 patients at approximately 1 patient for 2 controls. These patients were matched for age (±1 year), sex, and axial length (±1 mm).

Among cases, 12 eyes had normal tension glaucoma, 4 had primary open angle glaucoma, and 1 had secondary glaucoma due to sarcoidosis. Ten eyes had type 1 macular neovascularization (MNV), 1 had type 2 MNV, and 6 had polypoidal choroidal vasculopathy. The patients used PGAs (bimatoprost, travoprost, latanoprost, isopropyl unoprostone, and tafluprost), beta-blockers (carteolol hydrochloride and timolol maleate), CAIs (brinzolamide and dorzolamide hydrochloride), and/or alpha-2 agonist (brimonidine tartrate). Among the 17 patients, 10 patients used a single eye drop (7 used a PGA [3 used tafluprost, 2 used latanoprost, 1 used travoprost, and 1 used isopropyl unoprostone] and the remaining 3 used a CAI [dorzolamide hydrochloride], beta-blocker [carteolol hydrochloride], or alpha-2 agonist [brimonidine tartrate]); 2 patients used a PGA and a beta-blocker (tafluprost/timolol fixed-dose or travoprost/timolol fixed-dose); 2 patients used a PGA and an alpha-2 agonist (latanoprost and brimonidine tartrate); 1 patient used a CAI and a beta-blocker (brinzolamide/timolol fixed-dose) and an alpha-2 agonist (brimonidine tartrate); 1 patient used a CAI and a beta-blocker (brinzolamide/timolol fixed-dose) and a PGA (bimatoprost); and 1 used a CAI and a beta-blocker (dorzolamide hydrochloride/timolol), an alpha-2 agonist (brimonidine tartrate), and a PGA (bimatoprost).

Among the 40 controls, 12 had type 1 MNV, 2 had type 2 MNV, 3 had type 3 MNV, and 23 had polypoidal choroidal vasculopathy.

### Statistical Analysis

Patients were classified as PGA eye drop users, beta-blocker eye drop users, CAI eye drop users, and alpha-2 agonist eye drop users. Seven patients used a PGA alone and one patient each used a CAI, beta-blocker, or alpha-2 agonist alone. To evaluate the effect of eye drops that inhibit AH secretion on pharmacokinetics after intravitreal injection, patients who used more than one type of eye drop were analyzed in duplicate as users of either type. The drugs were categorized into two groups based on their mechanism of action on the AH: outflow drugs (e.g. PGA) and inflow drugs (e.g. CAI, beta-blockers, and alpha-2 agonists). For the purpose of this analysis, we sought to isolate drugs that exhibited purely outflow effects and classified them as “outflow drugs.” Therefore, alpha-2 agonists, which possess additional pharmacological actions beyond outflow effects, were not included in this category. Thus, for example, a patient who used prostaglandin, beta-blocker, and CAI eye drops was analyzed as an outflow drug user and as an inflow drug user. Aflibercept levels were compared between controls and patients using the using the Kruskal-Wallis test and Dunn's test. To further clarify the association between aflibercept levels and the type of eye drop used, multivariate analysis was conducted, including parameters such as age, sex, axial length, and time from aflibercept injection to AH sampling. To address the small sample size, we performed multivariate analyses using two models with a reduced set of independent variables. Categorical data were assessed using Pearson's chi-square test or Fisher's direct probability test. All statistical analyses were performed using JMP Pro software version 17.0.0 (SAS Institute Inc, Cary, NC). A *P* value < 0.05 was considered statistically significant. Results are reported as the mean (interquartile range [IQR]).

### Predicted Biological Activity

A previous report showed that the biological activity of VEGF-Trap was maintained for 79 to 87 days.^10^ Based on this result, we set the biological activity of aflibercept at an average of 84 days. The following formula determines the extension of aflibercept’s efficacy period through the adjunctive use of eye drops.

$$\begin{array}{c} DAYS=\frac{84\times \log\left(\frac{c^{A}28}{A_{o}}\right)}{\log\left(\frac{s^{A}28}{A_{o}}\right)} \end{array}$$

cA: Aflibercept level in the AH in controls, µg/mL.
sA: Aflibercept level in the AH in patients, µg/mL.
A_0_: Aflibercept level in the AH after aflibercept injection, µg/mL.
A_28_: Aflibercept level in the AH 28 days after aflibercept injection, µg/mL.
T_1/2_: The half-life time of aflibercept.

## Results

### Patient Demographics

Patients’ characteristics are shown in Table 1. The mean (IQR) ages of the controls, outflow drugs users, and inflow drugs users were 73.6 (IQR = 70.3–78.0) years, 71.3 (IQR = 65.0–77.0) years, and 76.1 (IQR = 74.8–80.0), respectively. The mean (IQR) times from aflibercept injection to AH sampling in the controls, outflow drugs users, and inflow drugs users were 29.9 (IQR = 29.0–30.8) days, 29.1 (IQR = 28.0–29.0) days, and 29.3 (IQR = 28.0–30.3), respectively. There was no significant difference in intraocular pressure between controls and patients (*P* = 0.78). All patients had consistently used glaucoma eye drops for at least 1 year.

**Table 1.** Characteristics of the Patients With Neovascular Age-Related Macular Degeneration in This Study

		Patients		
	Controls *N* = 40	Outflow Drugs *N* = 7	Inflow Drugs *N* = 10	*P* Value
Age, y, mean (IQR)^a^	73.6 (70.3–78.0)	71.3 (65.0–77.0)	76.1 (74.8–80.0)	0.24
Sex, male (%)^b^	21 (53)	5 (71)	3 (30)	0.23
Axial length, mm (IQR)^a^	23.83 (22.93–24.35)	23.85 (22.39–25.53)	23.73 (23.04–24.46)	0.99
IOP, mm Hg (IQR)^a^	14 (12–16)	13 (10–16)	15 (11–17)	0.78
Time from IVA to aqueous humor sampling, d (IQR)^a^	29.9 (29.0–30.8)	29.1 (28.0–29.0)	29.3 (28.0–30.3)	0.40
Subtype^b^^,^^c^	12, 2, 3, 23	4, 0, 0, 3	6, 1, 0, 3	0.43
Phakia (%)^b^	27 (68)	6 (83)	6 (60)	0.52

IOP, intraocular pressure; IQR, interquartile range; IVA, intravitreal aflibercept; mm Hg, millimeters of mercury.

a Kruskal-Wallis test.

b Pearson chi-square test.

c Subtype: macular neovascularization (MNV) type 1, MNV type 2, MNV type 3, and polypoidal choroidal vasculopathy.

The characteristics of controls and patients by eye drops used are shown in Supplementary Table S1. There was no significant difference in the time from aflibercept injection to AH sampling between controls and patients (*P =* 0.18) or specifically among patients using a CAI, beta-blocker, or alpha-2 agonist (*P* = 0.60, 0.60, and 0.80, respectively), but the time was significantly shorter in patients who used a PGA (*P* = 0.021). Although no outliers were identified, the distributions of the data differed, suggesting a statistically significant difference. Axial length was not significantly different among controls, outflow drugs users, and inflow drugs users (*P* = 0.99) or specifically among patients who used any eye drop (*P* = 0.71, 0.70, 0.71, and 0.49, for PGA, CAI, beta-blocker, and alpha-2 agonist, respectively).

### Aflibercept Level

**Figure 1.**
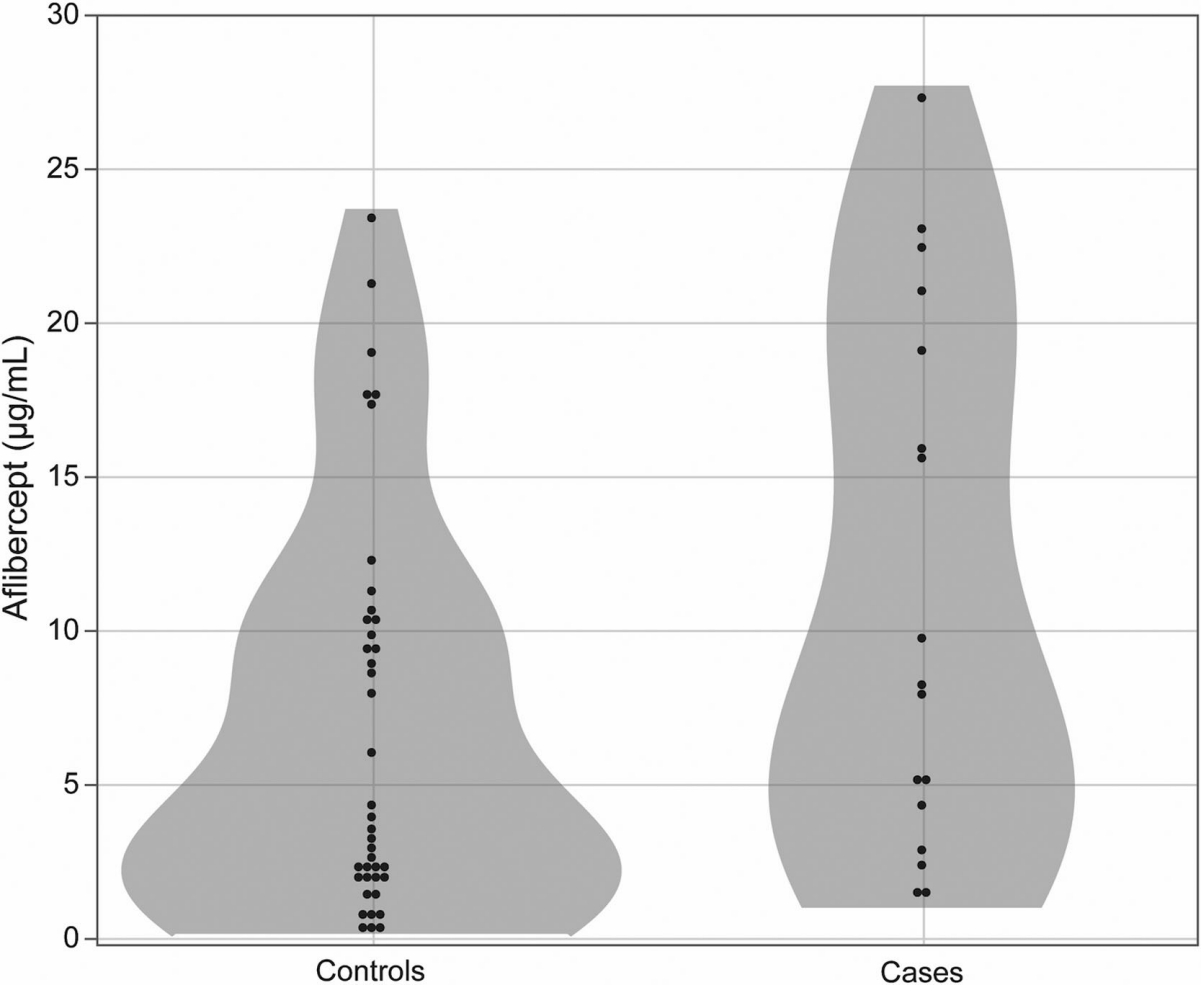
**Distribution of aflibercept levels in the aqueous humor.**  Wilcoxon rank-sum test showed that there was no significant difference between healthy controls and patients (*P* = 0.053).

**Figure 2.**
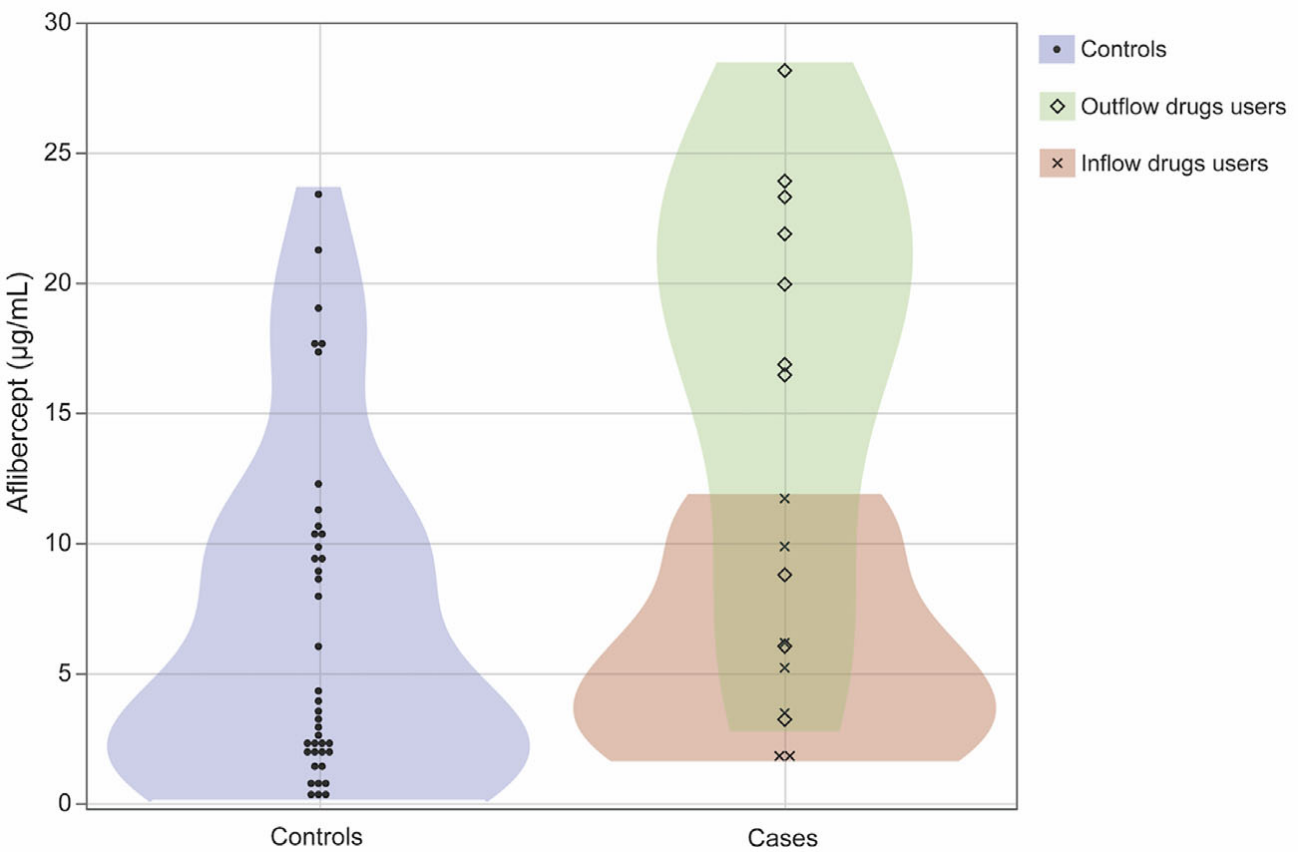
**Distribution of aflibercept levels in the aqueous humor for users of each type of eye drop.** Scatterplot of the aflibercept level in controls and patients who used eyed drops including outflow drugs (*green marker*) and inflow drugs (*red markers*). A Kruskal-Wallis test showed a significant difference among the control, inflow, and outflow drugs (*P* = 0.0075). Dunn’s test showed that aflibercept levels in the aqueous humor were significantly higher in cases using inflow drugs compared to both controls and patients using outflow drugs (*P* = 0.0085 and 0.044, respectively).

The distribution of aflibercept levels in controls and patients is illustrated in Figure 1. Although the aflibercept level in the AH was slightly higher in patients than in controls, there was no significant difference between controls and patients (*P* = 0.053). Figure 2 shows the distribution of aflibercept levels in controls and patients using each eye drop type.

Table 2 shows the mean (IQR) aflibercept levels in the AH in controls, outflow drug users, and inflow drug users: 6.83 (IQR = 1.94–10.34), 4.71 (IQR = 1.44–8.17), and 15.95 (IQR = 7.20–22.57) µg/mL, respectively. A Kruskal-Wallis test showed a significant difference among the control, inflow, and outflow drugs (*P* = 0.0075). Dunn’s test showed that aflibercept levels in the AH were significantly higher in patients using inflow drugs compared to both controls and patients using outflow drugs (*P* = 0.0085 and 0.044, respectively).

**Table 2.** Multivariate Associations Between Aflibercept Levels and Glaucoma Eye Drop Use

					Multivariate Adjusted			
			Univariate		Model 1^b^		Model 2^c^	
	*n*	Aflibercept, µg/mg	Mean Difference (95% CI)	*P* Value^a^	Mean Difference (95% CI)	*P* Value	Mean Difference (95% CI)	*P* Value
Control	40	6.83 (1.94 to 10.34)	Ref	Ref	Ref	Ref	Ref	Ref
Outflow drugs	7	4.71 (1.44 to 8.17)	6.78 (−2.89 to 16.44)	0.99	2.89 (−1.36 to 7.13)	0.19	1.59 (−2.53 to 5.71)	0.45
Inflow drugs	10	15.95 (7.20 to 22.57)	15.19 (5.08 to 25.28)	**0** **.0085**	8.36 (3.37 to 13.34)	**0** **.0020**	8.08 (3.22 to 12.94)	**0** **.0021**

Outflow drugs, prostaglandin analog; inflow drugs, beta-blocker, alpha-2 agonist, and carbonic anhydrase inhibitor.

The *P* values in bold represent statistical significance.

a Dunn's test (compared with controls).

b Model 1: adjusted for age, sex, and axial length.

c Model 2: adjusted for age, sex, axial length, and time from intravitreal aflibercept injection to aqueous humor sampling.

95% CI, 95% confidence interval.

Multiple regression analysis model 1, adjusted for the parameters age, sex, and axial length, showed that inflow drug use (CAI, beta-blocker, and alpha-2 agonist) was significantly associated with a high aflibercept level (*P* = 0.0020). Multiple regression analysis model 2, adjusted for the parameters age, sex, axial length, and time from intravitreal aflibercept (IVA) injection to AH sampling, also showed that inflow drug use was significantly associated with a high aflibercept level (*P* = 0.0021; see Table 2).

### Predicted Biological Activity

With a reported half-life of 9.3 to 11 days for aflibercept,^11^^–^^13^ our model estimates that the concentration will remain at or above the cA84 level for 144 to 166 days, indicating a potential 60 to 80 day extension of efficacy.

## Discussion

This study showed that aflibercept levels in the AH 1 month after the first aflibercept injection were higher in inflow drug users (drops including a CAI, beta-blocker, and alpha-2 agonist, *P* = 0.0085) than in controls. Multivariate analysis models 1 and 2 revealed that inflow drug users were significantly associated with a high aflibercept level (*P* = 0.0020 and 0.0021, respectively). These eye drops extended the duration of the biological activity of aflibercept by approximately 60 to 80 days.

CAIs, beta-blockers, and alpha-2 agonists are widely used to lower intraocular pressure by reducing AH secretion, whereas PGAs and alpha-2 agonists are also used to reduce resistance to AH outflow. Patients who used inflow drugs had a higher aflibercept level in the AH. Although the time from aflibercept injection to AH sampling was significantly shorter in PGA and outflow drug users, patients who used an outflow drug did not have a higher aflibercept level in the AH. Multivariate analysis demonstrated a significant association between inflow drug use and higher aflibercept levels in the AH, regardless of the time from aflibercept injection to AH sampling. The primary mechanism of AH outflow is pressure-dependent. The outflow drugs can alleviate impaired outflow in glaucoma but have minimal effects on AH turnover. In contrast, inflow drugs lower intraocular pressure and slow AH turnover, potentially delaying the elimination of anti-VEGF agents from the human eye. Although the anatomy of the rabbit outflow apparatus differs significantly from that of humans, a previous report using rabbit eyes demonstrated that brinzolamide and maleate fixed-combination eye drops significantly extended the ocular residence time of intravitreally injected aflibercept.^14^ The present study demonstrates the potential clinical use of topical eye drops to extend the ocular half-life of anti-VEGF agents.

It is difficult to determine the exact vitreous pharmacokinetics of anti-VEGF drugs. In this study, the vitreous pharmacokinetics of anti-VEGF drugs were based on the notion that anti-VEGF drugs passively diffuse into the AH and are released into the systemic circulation. When an inhibitor of AH secretion is used, the slower dilution rate of the drug concentration reaching the AH results in a higher concentration in the AH. Consequently, the vitreous-aqueous concentration gradient is expected to decrease, which may also affect the half-life in the vitreous. Previous reports have shown that the one-compartment model explains the measured values.^15^^,^^16^ This model supports the possibility that a slower aqueous secretion might lead to slower vitreous fluid turnover. However, there are still many factors that might affect the vitreous pharmacokinetics of drugs, such as how eye movements like saccades affect the convective-diffusive behaviors of intravitreally delivered drugs,^17^^–^^19^ and the retinal-choroid-sclera pathway.^20^^,^^21^ The mechanism discussed in the current study needs to be confirmed experimentally or by simulation using a model.^22^^–^^25^

This study has several limitations. Although the study period was 7 years, a relatively small number of cases from 2 institutions were analyzed. Approximately only 3% of Japanese patients have already started glaucoma treatment when they start nAMD treatment.^26^ The sample size of this study was limited by the inclusion criteria. Second, all cases were patients with glaucoma and might have an altered AH circulation. We attempted to perform a sensitivity analysis for patients after excluding those who used one or more eye drops, and there was one patient who used each type of eye drop except PGA. Because seven patients used multiple eye drops, the impact of a single type of eye drop on the AH could not be accurately assessed. Given that eye drops used to lower intraocular pressure have different mechanisms of action, either by reducing AH production or decreasing outflow resistance, we opted not to analyze the data based on eye drop classes. We conducted multivariate analysis to account for the effects of the use of multiple eye drops and it was challenging to fully assess the impact of each individual eye drop. Additionally, this study evaluated only the aflibercept level in the AH. Although the results showed that inhibition of AH secretion via eye drops extended the half-life of the intravitreally injected drugs, it is not clear whether this concentration difference affected the clinical outcome. Finally, all cases and controls were Japanese people and the study might be influenced by regional differences among the Japanese population. Further study including a focus on clinical outcomes is needed.

This study showed that aflibercept levels in the AH 1 month after the first aflibercept injection were higher in patients who used eye drops including a CAI, beta-blocker, or alpha-2 agonist than in healthy controls. Our findings suggest that decreasing AH secretion might extend the half-life of intravitreally injected drugs. The combination of these eye drops might reduce the number of injections needed for patients requiring intravitreal injections of drugs.

## Supplementary Material

Supplement 1
