# Supplementary material for: Effect of Combination Use of Aqueous Humor Secretion Inhibitor Eye Drops on Aflibercept Level: A Preliminary Analysis
Source: Transl Vis Sci Technol. 2025 Feb 20;14(2):21. doi: 10.1167/tvst.14.2.21 (PMC11844225; doi:10.1167/tvst.14.2.21)
Supplement: Supplement 1 [file tvst-14-2-21_s001.docx]

**Table S1. Patient characteristics based on type of eye drop used**

|  | **Controls**  **N = 40** | **Cases using eye drops including these agents** [*P*-value] | | | |
| --- | --- | --- | --- | --- | --- |
| Eye drops | N/A | PGA  n = 13 | CAI  n = 4 | Beta-blocker  n = 6 | Alpha-2 agonist  n = 5 |
| Age, y, mean (IQR)* | 73.6 (70.3-78.0) | 74.5 (69.5-80) [.49] | 73.3 (67.3-79) [.88] | 75 (73.5-77) [.50] | 77.2 (72-81) [.12] |
| Sex, male (%)^†^ | 21 (53) | 7 (54) [.81] | 0 (0) [**.035**] | 1 (17) [.077] | 2 (40) [.61] |
| Axial length, mm (IQR)* | 23.83 (22.93-24.35) | 23.83 (22.94-24.60) [.71] | 23.08 (23.32-24.04) [.70] | 23.59 (23.04-24.22) [.71] | 23.88 (23.64-24.19) [.49] |
| IOP, mmHg (IQR)* | 14 (12-16) | 13.6 (11-15) [.46] | 16.8 (13-20) [.18] | 13.8 (11-16) [.65] | 15.2 (13-18) [.44] |
| Time from IVA to aqueous humor sampling, d (IQR)* | 29.9 (29.0-30.8) | 28.5 (27.3-29.7) [**.021**] | 29.5 (24.7-34.3) [.59] | 29.2 (26.6-31.7) [.60] | 30.4 (25.7-35.1) [.80] |
| Subtype^†, ‡^ | 12, 2, 3, 23 | 7, 1, 0, 5 [.46] | 2, 0, 0, 2 [.90] | 3, 0, 0, 3 [.82] | 3, 1, 0, 1 [.24] |
| Phakia (%)^†^ | 27 (68) | 9 (69) [.94] | 3 (75) [.77] | 3 (50) [.30] | 3 (60) [.67] |

IOP, intraocular pressure; IQR, interquartile range; IVA, intravitreal aflibercept; PGA, prostaglandin analog; CAI, carbonic anhydrase inhibitor.

*: Wilcoxon rank-sum test compared with controls.

^†^: Pearson chi-square test.

^‡^: Subtype: macular neovascularization (MNV) type 1, MNV type 2, MNV type 3, and polypoidal choroidal vasculopathy.
